# Neutralization of the *Staphylococcus aureus* Panton-Valentine leukocidin by African and Caucasian sera

**DOI:** 10.1186/s12866-022-02636-5

**Published:** 2022-09-17

**Authors:** Tobias Grebe, Viktoria Rudolf, Christiane Sidonie Gouleu, Bettina Löffler, Ayola Akim Adegnika, Adebayo Osagie Shittu, Stefanie Deinhardt-Emmer, Silke Niemann, Frieder Schaumburg

**Affiliations:** 1 Institute of Medical Microbiology, University of Münster, Münster, Germany; 2 Centre de Recherches Médicales de Lambaréné, Lambaréné, Gabon; 3 Institut für Tropenmedizin, Universität Tübingen and German Center for Infection Research, Tübingen, Germany; 4 Institute of Medical Microbiology, Jena University Hospital, Jena, Germany; 5 Obafemi Awolowo University, Ile-Ife, Nigeria

**Keywords:** *Staphylococcus aureus*, Panton-Valentine leukocidin, Neutralization test, Polymorphonuclear leukocytes

## Abstract

**Background:**

The prevalence of *Staphylococcus aureus* isolates carrying the Panton-Valentine leukocidin (PVL) gene is higher in Africa (≈50%) compared to Europe (< 5%). The study aimed to measure anti-PVL-antibodies in Africans and Germans in a multi-center study and to test whether detected antibodies can neutralize the cytotoxic effect of PVL on polymorphonuclear leukocytes (PMNs).

**Methods:**

Sera from asymptomatic Africans (*n* = 22, Nigeria, Gabon) and Caucasians (*n* = 22, Germany) were used to quantify antibody titers against PVL and α-hemolysin (in arbitrary units [AU]) by ELISA. PMNs from one African and German donor were exposed to 5 nM recombinant PVL to measure the neutralizing effect of serial dilutions of pooled sera from African and Caucasian participants, or donor sera at 0.625 and 2.5% (v/v).

**Results:**

Anti-PVL-antibodies were significantly higher in Africans than in Germans (1.9 vs. 0.7 AU, *p* < 0.0001). The pooled sera from the study participants neutralized the cytotoxic effect of PVL on African and German PMNs in a dose dependent manner. Also, neutralization of PVL on PMNs from the African and German donors had a stronger effect with African sera (half-maximal inhibitory concentration (IC_50_) = 0.27 and 0.47%, respectively) compared to Caucasian sera (IC_50_ = 3.51 and 3.59% respectively).

**Conclusion:**

Africans have higher levels of neutralizing anti-PVL-antibodies. It remains unclear if or at what level these antibodies protect against PVL-related diseases.

**Supplementary Information:**

The online version contains supplementary material available at 10.1186/s12866-022-02636-5.

## Background

*S. aureus* encodes a wide variety of virulence factors and toxins, including hemolysins and leukocidins, to establish an infection and evade the host immune system [1, 2]. Single- and bi-component pore-forming toxins such as α-hemolysin (Hla) and the Panton-Valentine leukocidin (PVL), respectively, target the cell membrane of immune cells, resulting in their lysis [3]. PVL is primarily associated with severe skin and soft tissue infection (SSTI, e.g. pyomyositis) and most likely involved in the pathogenesis of necrotizing pneumonia [4–6]. After secretion, the LukS-PV and LukF-PV components of PVL, bind to their specific receptors, complement 5a receptor (C5aR) and CD45, on polymorphonuclear leukocytes (PMNs, i.e. neutrophil granulocytes), macrophages, and monocytes and hetero-oligomerize into an octameric pore to exert their cytotoxic effect [7, 8].

The prevalence of PVL-positive *S. aureus* varies significantly between geographic regions. In sub-Saharan Africa, up to 74% of methicillin-susceptible *S. aureus* isolates encode PVL genes [9], while this proportion is lower in Europe (0.2%) and the United States (11.5%; however, in methicillin-resistant *S. aureus* isolates [MRSA] the prevalence can be as high as 48.1%, mostly due to the epidemic spread of the PVL-positive MRSA clone USA 300) [10–12]. This appears to be of clinical relevance as the incidence of SSTI in infants is higher in sub-Saharan Africa compared to the US [13].

In addition, a multi-center study revealed that the titers of anti-PVL-antibodies correspond to the prevalence of PVL-positive *S. aureus* in the geographic regions. High anti-PVL-antibody levels were observed in a study population from Senegal (12.6 AU/ml) and low titers in participants from France (1.5 AU/ml) [14]. It remains unclear whether these anti-PVL-antibodies are protective in vivo.

Some methods are available to measure the neutralizing effect of anti-PVL-antibodies on PVL-mediated cytotoxicity or disease. They include rabbit models [15], three-dimensional tissue models [16], or ex-vivo human skin models [17]. However, these models are labor-intensive for routine testing. To assess the neutralization of PVL by immunoglobulins, whole blood or granulocyte assays have been suggested [18, 19].

The objectives of the study were to compare the anti-PVL-antibody titers among Africans and Caucasians in a multi-center study and evaluate PVL neutralization in a granulocyte-based assay.

## Results

The median age of Africans and Germans was comparable (37.5 vs. 35 years, Table 1). The proportion of females was higher in Germany compared to the African study sites (82% vs. 45%, *p* = 0.03, Table 1). Participants with a history of hospitalization, antimicrobial treatment, or skin and soft tissue infection in the past 6 months were only identified in the African group. No statistically significant difference in the colonization with *S. aureus* could be observed between African and German participants (27% vs. 14%, *p* = 0.46, Table 1).

**Table 1:** Demographic and clinical data of serum donors from Africa and Germany

	Germans (*n* = 22)	Africans (*n* = 22)	OR (95%CI)	*p* value
Age [median (range)]	35 (21–59)	37.5 (20–61)	NA	0.55
Female sex [% (n)]	82 (18)	45 (10)	0.19 (0.06–0.68)	0.03
Hospitalization in the past 6 months [% (n)]	0 (0)	5 (1)	NA	> 0.99
History of skin and soft tissue infection in the past 6 months [% (n)]	0 (0)	14 (3)	NA	0.23
Use of antimicrobial agents in the past 6 months [% (n)]	0 (0)	18 (4)	NA	0.11
Known HIV infection [% (n)]	0 (0)	0 (0)	NA	NA
Nasal *S. aureus* colonization [% (n)]	9 (2)	23 (5)	0.34 (0.06–2.04)	0.41
Pharyngeal *S. aureus* colonization [% (n)]	5 (1)	14 (3)	0.30 (0.02–2.23)	0.61
Any *S. aureus* colonization [% (n)]	3 (14)	6 (27)	0.42 (0.10–1.75	0.46

**Fig. 1:**
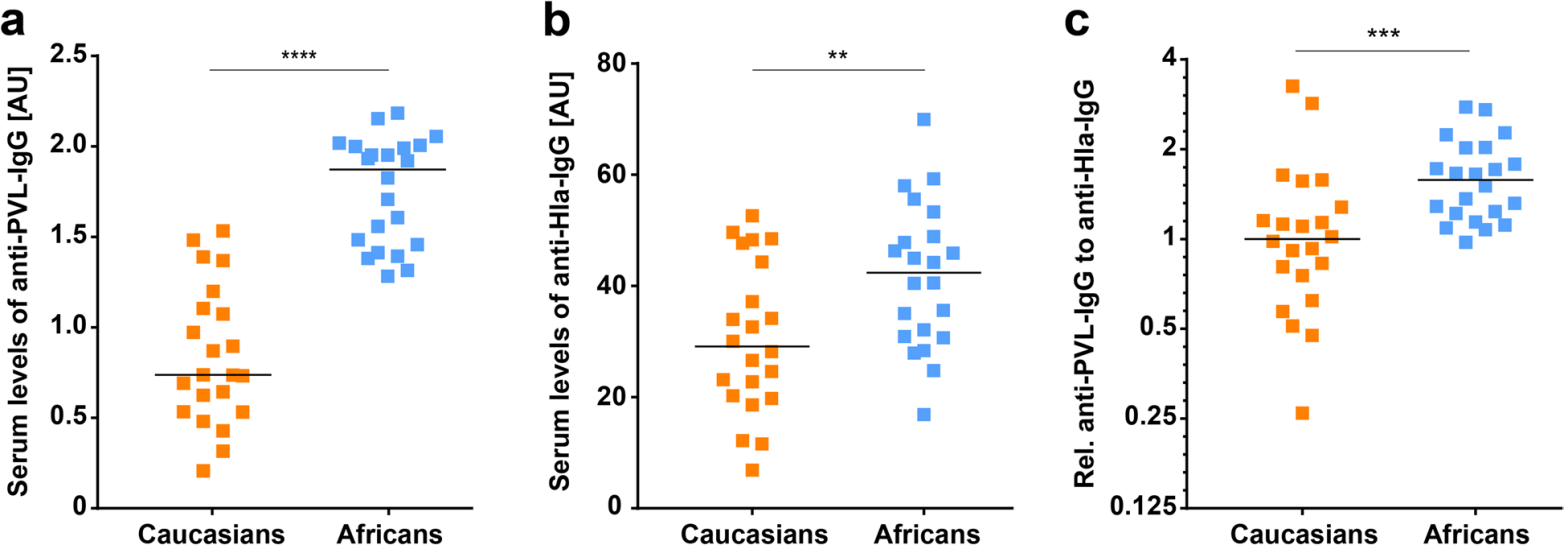
Anti-Panton-Valentine-leukocidin (PVL)-antibodies in Africans compared to Germans. Serum levels of anti-PVL-antibodies (**a**) and anti-Hla-antibodies (**b**) of participants from Germany and Africa were quantified by ELISA and expressed in arbitrary units (AU). Median values for levels of antibodies to PVL in Caucasians and Africans were 0.7 AU (interquartile range (IQR), 0.5–1.1 AU) and 1.9 AU (IQR, 1.4–2 AU), respectively. Median values for levels of antibodies to Hla in Caucasians and Africans were 29.1 AU (IQR, 20.1–40.2 AU) and 42.4 AU (IQR, 30.8–50 AU), respectively. **c** The titers of antibodies against PVL were corrected for their respective anti-Hla titers and displayed as fold-change to median serum level of Caucasians (IQR, 0.7–1.4). Median value for relative anti-PVL titers in Africans was 1.6 (IQR, 1.2–2). Statistical significance was analyzed by Mann-Whitney test or unpaired t test (** *p* < 0.01, *** *p* < 0.001, **** *p* < 0.0001)

First, we measured the anti-PVL-antibody titers in the sera of healthy participants and observed significantly higher levels in Africans than Germans (1.9 vs. 0.7 AU, *p* < 0.0001, Fig. 1a). To test for potential differences in exposure to *S. aureus* between the two groups we quantified serum levels of antibodies to Hla. Participants from Africa had higher levels compared to Germans (42.4 AU vs. 29.1 AU, *p* = 0.008, Fig. 1b). After normalization of anti-PVL to anti-Hla antibody titers, the levels against PVL remained significantly higher in Africans (1.6-fold, *p* = 0.0007, Fig. 1c).

**Fig. 2:**
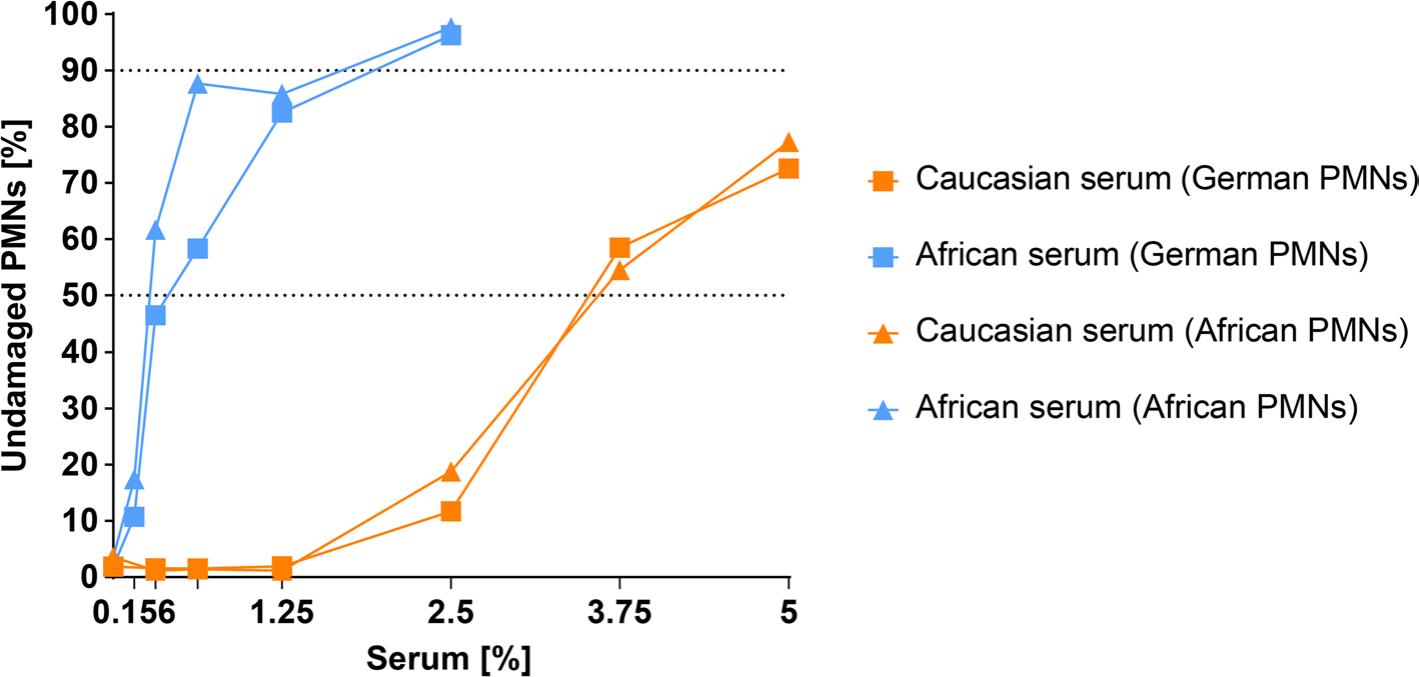
Isolated polymorphonuclear leukocytes (PMNs) from the German and African donor were subjected to 5 nM recombinant Panton-Valentine leukocidin (PVL) after pre-incubation with serial dilutions of pooled serum from German or African participants. Neutralization of the cytotoxic effect of PVL is displayed as percentage of undamaged PMNs. Half-maximal (50%) and 90% inhibition thresholds are indicated as dotted lines

Next, we analyzed the capacity of these antibodies to neutralize the cytotoxic effect of PVL on PMNs. Briefly, isolated PMNs of one African and German donor were treated with 5 nM recombinant PVL after pre-incubation with increasing concentrations of pooled sera from African (*n* = 22) or German (*n* = 22) participants. The pooled sera neutralized the cytotoxic effect of PVL on African and German PMNs in a dose-dependent manner (Fig. 2). Determination of the half-maximal inhibitory concentrations (IC_50_) showed that neutralization of the cytotoxic effect of PVL on PMNs from the African and German donors had a stronger effect with African sera (0.27 and 0.47%, respectively) than Caucasian sera (3.51 and 3.59%, respectively).

We next plotted the levels of anti-PVL-antibodies against the percentage of undamaged PMNs after PVL treatment with individual sera (at 0.625 and 2.5%) from African and German participants. It revealed a strong correlation between antibody titer and neutralization of PVL-induced cell damage (Fig. S1). Sera with comparable anti-PVL-antibody titers neutralized the cytotoxic effect of PVL to a similar extent. This was independent of whether the serum was from Africans or Germans.

## Discussion

We observed higher levels of anti-PVL-antibodies in the sera of Africans than Germans, with a corresponding higher in vitro capacity to neutralize the cytotoxic effect of PVL on human PMNs. Our results suggest that the higher humoral immune response against PVL in healthy adults from sub-Saharan Africa is indicative of the high prevalence of PVL-positive *S. aureus* in the African continent compared with Germany. Furthermore, the correlation of the inhibitory effect of serum on PVL-induced cell damage with the amount of quantified serum anti-PVL-antibodies revealed a linear relationship indicating a titer-dependent neutralization, which is similar to an earlier study [18].

To determine whether the differences in anti-PVL-antibody levels between Africans and Germans were due to different exposure to *S. aureus* instead of spatial differences in the prevalence of PVL-positive *S. aureus*, we measured serum levels of antibodies against Hla [14, 20]. The slightly elevated amount of antibodies against Hla in African participants compared to Germans may indicate a higher exposure to *S. aureus* in the African group. After normalization of anti-PVL to anti-Hla titers, there was still a significant difference between Africans and Germans. This suggests that the humoral response to PVL correlates with the local prevalence of PVL-positive *S. aureus* [14]. However, whether higher antibody levels are associated with protection in vivo remains unclear. It has recently been shown that antibody responses against PVL in the general population increase with age [20]. Given the higher incidence of SSTI in children in PVL-endemic sub-Saharan Africa [13, 21], it could be assumed that the immature humoral immunity of children lacks protective antibodies against PVL. Indeed, in a recent study on *S. aureus* pneumonia almost all cases in a patient cluster under the age of three were caused by PVL-positive *S. aureus* [22]. Nevertheless, Hermos et al. [23] reported that antibody levels against PVL were significantly elevated in children with PVL-positive methicillin-resistant *S. aureus* (MRSA) SSTI compared to children infected with PVL-negative MRSA or to uninfected children. Of note, the highest titers of anti-PVL-antibodies were present in children with a prior MRSA infection or SSTI compared to those without a history of infection. While these antibodies effectively neutralized the cytotoxic effect of PVL on PMNs in vitro, it appeared that high levels of anti-PVL-antibodies did not protect against PVL-associated SSTI [23]. Nonetheless, in a rabbit necrotizing pneumonia model, passive immunization with antibodies targeting PVL conferred protection against mortality when challenged with PVL-positive *S. aureus* MRSA strains [15, 24].

Our study has limitations. First, we cannot conclude that neutralizing antibodies in our leukocyte assay are protective in vivo. Therefore, prospective cohort studies with baseline antibody levels and prospective surveillance of PVL-related infections need to be performed. Second, the majority of human *S. aureus* isolates encode other leukocidins, i.e. γ-haemolysin AB (HlgAB) and CB (HlgCB), leukocidin ED (LukED) and leukocidin GH (LukGH, also known as LukAB) [3]. Due to the high homology of the bi-component toxins, cross-neutralization of PVL can be due to antibodies against other leukotoxins [24, 25]. Third, the small sample size per study site might not be representative of the serologic status of the entire regional population.

## Conclusion

In summary, this study shows that sera of African participants contained significantly higher titers of antibodies against PVL. Furthermore, neutralization of PVL with African serum was notably stronger compared to Caucasian sera, while the protective effect seems to be solely titer-dependent. This suggests that the high prevalence of PVL-positive *S. aureus* is reflected by a possible protective immune response. However, whether these antibodies protect against PVL-related diseases remains unclear.

## Materials and methods

### Study population

A convenience sample of 11 sera per study site was considered appropriate as described previously [19]. A specific sample size calculation was not performed.

All sera were obtained from healthy volunteers without signs or symptoms of *S. aureus* infection. Participants were screened for nasal and pharyngeal *S. aureus* colonization. Briefly, the mucous membranes of the anterior nares and throat were swabbed (Transwab Amies, Medical Wire, Corsham, UK) with light pressure and cultured on Columbia blood agar (BD, Heidelberg, Germany). *S. aureus* was confirmed by MALDI-TOF mass spectrometry (Bruker, Bremen, Germany). Demographic and clinical data (age, sex, hospitalization, use of antibiotics, history of skin and soft tissue infection, known HIV infections) were recorded in standard case report forms.

### Production and purification of PVL

The PVL subunits LukS-PV and LukF-PV were produced and purified as previously described [26]. In brief, lukS-PV and lukF-PV were recombinantly expressed from isopropyl-beta-D-thiogalactopyranoside (IPTG)-inducible pQE30UA in *Escherichia coli* TG1. The 6-His-tagged proteins were purified from cell lysates by nickel-nitrilotriacetic acid affinity resin (Qiagen, Hilden, Germany), and the buffer was exchanged with phosphate-buffered saline (PBS) utilizing PD-10 Sephadex G-25 medium columns (Cytiva, Marlborough, USA).

### Quantification of antibodies against PVL and α-hemolysin in the plasma

Levels of antibodies in the plasma against PVL or α-hemolysin (Hla) were determined using an ELISA method adapted from Niemann et al. [18]. Since Hla is produced by virtually all *S. aureus* isolates, anti-Hla-antibodies were measured to assess the overall exposure to *S. aureus* in the study population [14]. A microtiter plate (Nunc Maxisorb, ThermoFisher, Waltham, USA) was coated overnight at room temperature with 40 μg/ml recombinant LukS-PV and LukF-PV, or 1.5 μg/ml Hla (Sigma-Aldrich Merck KGaA, Darmstadt, Germany), respectively. After blocking with 10% (w/v) skimmed milk in PBS-T (0.05% Tween 20) for 1 h at 37 °C, unbound protein was removed by washing twice with PBS-T. Diluted plasma samples (1:2500 in PBS) were added as duplicates and incubated for 1 h at 37 °C. Following four washing steps with PBS-T, horseradish peroxidase (HRP)-conjugated goat-anti-human-IgG antibody (1:5000, Promega, Madison, USA) was added and the microtiter plates were incubated for 1 h at 37 °C. After another four washing steps, the peroxidase substrate (SIGMAFAST ODP tablet set, Sigma-Aldrich) was added, and the plates were incubated for 30 minutes at room temperature in the dark. Absorbance was measured at 450 nm on a microplate reader (iMark, Bio-Rad Laboratories, München, Germany) and the results were expressed in arbitrary units (AU). To control for overall higher exposure to *S. aureus* in one group, we normalized the levels of anti-PVL-antibodies against those of anti-Hla-antibodies.

### Preparation of human polymorphonuclear leukocytes (PMNs)

Human PMNs were freshly isolated from sodium citrate (3.2%) blood of healthy donors. After dextran-sedimentation, density gradient centrifugation using Ficoll-Paque Plus (Sigma-Aldrich) was performed according to the manufacturer’s protocol. Subsequently, hypotonic lysis of remaining erythrocytes was performed by a 20-s incubation in sterile water and stopped by adding an equal volume of 1.8% sodium chloride solution. As the last step, PMNs were resuspended in RPMI-1640 culture medium (Sigma-Aldrich) at a final concentration of 1 × 10^6^ cells/ml and directly used for the experiments.

### Neutralization assay

Freshly prepared human PMNs were pre-incubated with serum in the indicated concentrations (final concentration, 0.156–5%) for 30 min at room temperature. Subsequently, 5 nM recombinant LukS-PV and LukF-PV were added and incubated for 1 h at room temperature with shaking at 5 rpm. To measure PVL-induced cell damage, samples were stained with 5 μg/ml propidium iodide (Sigma-Aldrich) and analyzed using an Accuri C6 Flow cytometer (BD). A total of 5000 gated events were analyzed for each sample. The neutralizing effect of serum against PVL-induced cell damage was calculated from duplicate experiments as the percentage of undamaged PMNs compared to the untreated control without serum.

### Statistical analysis

All statistical analyses were performed using Prism (GraphPad Software, San Diego, USA). Differences between Africans and Caucasians were tested using the Mann-Whitney test (not normally distributed data) or unpaired t-test (normally distributed data). The correlation between anti-PVL-antibodies and neutralization capacity was determined by computing the Spearman correlation coefficient and linear regression.

## Supplementary Information

**Additional file 1: Supplementary Fig. S1.** Correlation of antibodies against Panton-Valentine leukocidin (PVL) with the neutralizing effect on PVL-induced cell damage. Serum levels of anti-PVL-antibodies are plotted against the amount of undamaged polymorphonuclear leukocytes (PMNs) from the African or German donor after treatment with 5 nM recombinant PVL in the presence of 0.625% or 2.5% serum from African (blue triangles) or Caucasian (orange circles) participants. Linear regression and correlation analyses of a given population (color-coding) are indicated as the coefficient of determination (R^2^) and Spearman’s correlation test coefficients (r) with probability (p), respectively.

## Data Availability

The datasets used and/or analyzed during the current study are available from the corresponding author on reasonable request.

## Abbreviations

PVL: Panton-Valentine leukocidin
PMNs: Polymorphonuclear leukocytes
Hla: Alpha-hemolysin
