# Supplementary material for: Neutralization of the Staphylococcus aureus Panton-Valentine leukocidin by African and Caucasian sera
Source: BMC Microbiol. 2022 Sep 17;22:219. doi: 10.1186/s12866-022-02636-5 (PMC9482280; doi:10.1186/s12866-022-02636-5)
Supplement: Supplementary file 1 — Additional file 1: Supplementary Fig. S1. Correlation of antibodies against Panton-Valentine leukocidin (PVL) with the neutralizing effect on PVL-induced cell damage. Serum levels of anti-PVL-antibodies are plotted against the amount of undamaged polymorphonuclear leukocytes (PMNs) from the African or German donor after treatment with 5 nM recombinant PVL in the presence of 0.625% or 2.5% serum from African (blue triangles) or Caucasian (orange circles) participants. Linear regression and correlation analyses of a given population (color-coding) are indicated as the coefficient of determination (R2) and Spearman’s correlation test coefficients (r) with probability (p), respectively. [file 12866_2022_2636_MOESM1_ESM.docx]

**Supplementary material**


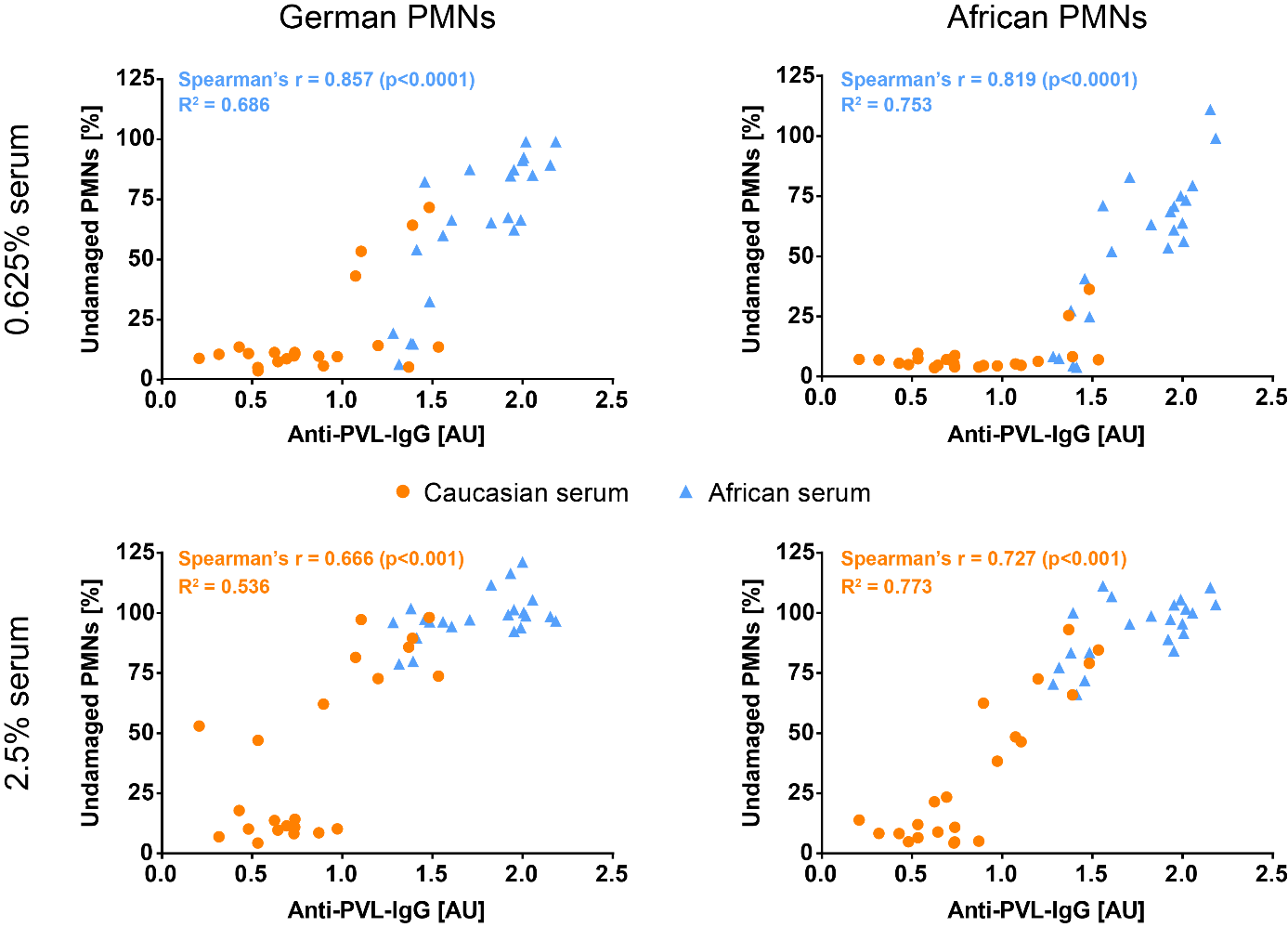


Supplementary Figure S1: Correlation of antibodies against Panton-Valentine leukocidin (PVL) with the neutralizing effect on PVL-induced cell damage. Serum levels of anti-PVL-antibodies are plotted against the amount of undamaged polymorphonuclear leukocytes (PMNs) from the African or German donor after treatment with 5 nM recombinant PVL in the presence of 0.625% or 2.5% serum from African (blue triangles) or Caucasian (orange circles) participants. Linear regression and correlation analyses of a given population (color-coding) are indicated as the coefficient of determination (R^2^) and Spearman’s correlation test coefficients (r) with probability (p), respectively.
